# Comparison of methods for estimating the attributable risk in the context of survival analysis

**DOI:** 10.1186/s12874-016-0285-1

**Published:** 2017-01-23

**Authors:** Malamine Gassama, Jacques Bénichou, Laureen Dartois, Anne C. M. Thiébaut

**Affiliations:** 1 Université Paris-Saclay, Biostatistics, Biomathematics, Pharmacoepidemiology and Infectious Diseases (B2PHI), Inserm, UVSQ, Institut Pasteur, 25 rue du Dr. Roux, Paris Cedex 15, 75724 France; 2 University of Rouen, Inserm, U 1219, 1 rue de Germont, Rouen Cedex, 76031 France; 3 Rouen University Hospital, Department of Biostatistics, 1 rue de Germont, Rouen Cedex, 76031 France; 4 Université Paris-Saclay, CESP, Fac. de médecine - Univ. Paris-Sud, Fac. de médecine - UVSQ, INSERM, 114 rue Edouard Vaillant, Villejuif Cedex, 94805 France; 5 Gustave Roussy, 114 rue Edouard Vaillant, Villejuif Cedex, 94805 France

**Keywords:** Attributable risk, Weighted Kaplan-Meier estimator, Piecewise constant hazards model, Cox model, Cohort studies, Breast cancer

## Abstract

**Background:**

The attributable risk (AR) measures the proportion of disease cases that can be attributed to an exposure in the population. Several definitions and estimation methods have been proposed for survival data.

**Methods:**

Using simulations, we compared four methods for estimating AR defined in terms of survival functions: two nonparametric methods based on Kaplan-Meier’s estimator, one semiparametric based on Cox’s model, and one parametric based on the piecewise constant hazards model, as well as one simpler method based on estimated exposure prevalence at baseline and Cox’s model hazard ratio. We considered a fixed binary exposure with varying exposure probabilities and strengths of association, and generated event times from a proportional hazards model with constant or monotonic (decreasing or increasing) Weibull baseline hazard, as well as from a nonproportional hazards model. We simulated 1,000 independent samples of size 1,000 or 10,000. The methods were compared in terms of mean bias, mean estimated standard error, empirical standard deviation and 95% confidence interval coverage probability at four equally spaced time points.

**Results:**

Under proportional hazards, all five methods yielded unbiased results regardless of sample size. Nonparametric methods displayed greater variability than other approaches. All methods showed satisfactory coverage except for nonparametric methods at the end of follow-up for a sample size of 1,000 especially. With nonproportional hazards, nonparametric methods yielded similar results to those under proportional hazards, whereas semiparametric and parametric approaches that both relied on the proportional hazards assumption performed poorly. These methods were applied to estimate the AR of breast cancer due to menopausal hormone therapy in 38,359 women of the E3N cohort.

**Conclusion:**

In practice, our study suggests to use the semiparametric or parametric approaches to estimate AR as a function of time in cohort studies if the proportional hazards assumption appears appropriate.

**Electronic supplementary material:**

The online version of this article (doi:10.1186/s12874-016-0285-1) contains supplementary material, which is available to authorized users.

## Background

In epidemiology, it is important not only to assess the association between one exposure and the occurrence of health events, but also to quantify the impact of this exposure on the occurrence of these events. This is done by estimating the attributable risk (AR) or the proportion of cases associated with this exposure in the population. This estimation takes into account not only the strength of the link between exposure and disease but also the importance (prevalence) of exposure in the population [1]. It expresses the proportion of disease cases that can be attributed to exposure [2], that is to say, under certain conditions, the proportion of potentially preventable cases by eliminating exposure. The AR is defined as:

1 $$ AR=\frac{\mathbf{P}(D) - {\mathbf{P}}(D|\bar{E})}{\mathbf{P}(D)},  $$

where **P**(*D*) is the probability of disease (incidence) in the population, which includes exposed *E* and unexposed $\bar {E}$ subjects, and $\mathbf {P}(D | \bar {E})$ is the hypothetical probability of disease in the same population but with all exposure eliminated.

The AR can be estimated from different types of studies including case-control studies for which many estimation methods exist (as reviewed in [3]), but it is rarely estimated from cohort studies. In the context of cohort studies and time-to-event outcomes, AR measures can be defined as functions of time [4-9] although a single AR estimate has been proposed alternatively [10].

Recent developments for estimating AR as a function of time from cohort studies in the survival analysis context have not so far led to a consensus definition. Several definitions have been proposed depending on whether authors interpret disease incidences **P**(*D*) and $\mathbf {P}(D|\bar {E})$ in Eq. (1) as cumulative distribution functions (CDFs) [6-9] or as instantaneous hazard functions [4, 5]. The two definitions converge only for rare diseases or low exposure prevalence [4]. Here we focus on the first definition of AR based on CDFs which looks more consistent with the standard AR definition and appears to be the most used in the literature. Several methods of estimation have been proposed for the AR defined in this case, including nonparametric approaches based on Kaplan-Meier’s estimator of the survival function [7], a semiparametric approach based on Cox’s proportional hazards model [7] and a fully parametric approach assuming a piecewise constant hazards model [8]. Some evaluations were made for the nonparametric and semiparametric approaches [7] but, to the best of our knowledge, the performances of these various approaches have not been systematically compared.

The aim of this paper was to compare available methods for estimating AR when defined using CDFs. In the sections to follow, we first review the corresponding estimation methods so far published in the statistical literature. Simulations were conducted to assess the performance of the proposed AR estimators. The methods were then applied to data on menopausal hormone therapy (MHT) and breast cancer from the E3N women cohort (*Étude Épidémiologique auprès de Femmes de la Mutuelle Générale de l’Éducation Nationale*) [11]. For the purpose of our illustration, we considered 38,359 participants who were postmenopausal and free of cancer when they completed a self-administered questionnaire on their past use of any MHT in January 1992. In total, 17,185 (44.8*%*) women had ever used MHT at baseline and were considered exposed thereafter. By June 2008 (for a maximal 16.4 years and mean 14.0 years of follow-up), 2,228 invasive breast cancers had been diagnosed (1, 106 in unexposed women). A recent work on the E3N cohort estimated a 14.5% postmenopausal breast cancer risk attributable to MHT use after 15 years of follow-up [12]. We estimated AR as a CDF-based function of time at four time points using nonparametric, semiparametric and parametric approaches, as well as the single overall AR measure proposed by Spiegelman et al. [10].

## Methods

### Review of estimation methods

When interpreting the incidence of disease **P**(*D*) as the event probability until some time *t*, the AR is defined as follows [4, 6, 7]:

$$ A(t) = \frac{\mathbf{P}(T \leq t) - \mathbf{P}(T \leq t | Z=z^{*})}{\mathbf{P}(T \leq t)} $$

where *T* denotes the time to disease or event time, *Z* a *p*-vector of risk factors and *z* ^∗^ the *p*-vector of their chosen target values in order to quantify the potential impact of modifying the current distribution of *Z* to *z* ^∗^. Since, in most applications, *z* ^∗^ is defined by setting one of the components of *Z* to its baseline (unexposed) level, we use notation *Z*=0 instead of *Z*=*z* ^∗^ in the following. Using the survival functions *S*(*t*)=**P**(*T*>*t*) and *S*_0_(*t*)=*S*(*T*>*t*|*Z*=0), the AR for time-to-event outcomes can be written as follows [7, 9]:

2 $$ A(t)=\frac{S_{0}(t)-S(t)}{1-S(t)}=1-\frac{1-S_{0}(t)}{1-S(t)}.  $$

A natural estimate of *A*(*t*) is obtained by replacing the survival functions *S*_0_(.) and *S*(.) by their respective estimators $\hat {S}_{0}(.)$ and $\hat {S}(.)$. Various estimators $\hat {S}_{0}(.)$ and $\hat {S}(.)$ have been proposed, as detailed in the following subsubsections.

#### Nonparametric approaches

Chen et al. [7] considered several approaches for estimating survival functions *S*_0_(.) and *S*(.) depending on covariate type: nonparametric when all *p* covariates are categorical and independent of time, otherwise semiparametric. The former case applies to a single categorical covariate or several covariates forming *K*+1 categories.

When all *p* covariates are categorical and independent of time and under the assumption that censoring is independent of the covariates, Chen et al. [7] suggested estimating both *S*_0_(.) and *S*(.) by the Kaplan-Meier method [13].

When all *p* covariates are categorical and independent of time but the assumption of covariate-independent censoring does not hold, Chen et al. [7] suggested estimating *S*(.) by the weighed Kaplan-Meier (WKM) estimator [14] and *S*_0_(.) by the Kaplan-Meier method. For a *p*-vector *Z* of covariates with *K*+1 categories, the WKM estimator is defined as:

$$ \hat{S}(t)=\frac{1}{n}\sum\limits_{k=0}^{K} n_{k} \hat{S}_{k}(t) $$

where $\hat {S}_{k}(t)$ is the Kaplan-Meier estimator among those with covariate profile *k*=0,1,2,…,*K* and *n*_*k*_ is the number of subjects with covariate profile *k* so that $\sum _{k=0}^{K} n_{k} $ equals *n*, the total number of subjects.

In all cases, the estimation of the variance of $\hat {A}(t)$ is based on the expression of $\{\hat {A}(t) - A(t)\}$ as a linear combination of $\{\hat {S}_{0}(t) - S_{0}(t)\}$ and $\{\hat {S}(t) - S(t)\}$ and relies on counting process results [7].

#### Semiparametric approach

For a more general type of covariates *Z*, i.e., when covariates are continuous, time-dependent or with too large a number of profile categories for nonparametric approaches, Chen et al. [7] considered using semiparametric instead of nonparametric methods to estimate *S*_0_(.) and *S*(.). Of these, the Cox proportional hazards model [15] is one of the most familiar. It assumes that, at any time *t*, the hazard function *λ*(*t*|*Z*) is the product of a nonparametric baseline hazard *λ*_0_(*t*) and a parametric function of the *p*-vector of covariates *Z* (or *Z*(*t*) in the case of time-dependent covariates) and the *p*-vector of corresponding parameters *β*. The parametric function is usually taken to be the exponential function, such that *λ*(*t*|*Z*)=*λ*_0_(*t*) exp(*β* ^*T*^ *Z*). In this case,

$$\begin{aligned} \hat{S}_{0}(t)&=\exp\!\left[-\hat{\Lambda}_{0}(t)\right] \text{~and~}\\ \hat{S}(t)&=\frac{1}{n}\sum\limits_{i=1}^{n}\exp\!\left[-{\int\limits_{0}^{t}}\exp\{\hat{\beta}^{T}z_{i}(u)\}d\hat{\Lambda}_{0}(u)\right] \end{aligned} $$

where $\hat {\Lambda }_{0}(.)$ is the Breslow estimator [16] of the baseline cumulative risk $\Lambda _{0}(t) = {\int \limits _{0}^{t}} \lambda _{0}(u) du$ and $\hat {\beta }$ is the maximum partial likelihood estimator.

The expression of the variance of $\hat {A}(t)$ follows the same general principles as for the nonparametric approaches above [7].

#### Parametric approach

Laaksonen et al. [8] proposed a parametric estimator based on a proportional hazards model with piecewise constant hazards (PCH). In this approach, follow-up time is partitioned into *J* prespecified intervals (0=*a*_0_,*a*_1_],(*a*_1_,*a*_2_],…,(*a*_*j*−1_,*a*_*j*_],…,(*a*_*J*−1_,*a*_*J*_], and the survival function at time *t* is estimated assuming a constant baseline hazard $\hat {\lambda }_{0j} = \exp (\hat {\alpha }_{j})$ in each *j*-th interval (*a*_*j*−1_,*a*_*j*_], *j*=1,2,…,*J* as follows:

$$ \hat{S}_{PCH}(t|Z_{i}) = \exp\!\left\{-\sum_{j=1}^{J} \exp(\hat{\alpha}_{j}+\hat{\beta}^{T}Z_{i})\delta_{j}(t)\right\} $$

where *δ*_*j*_(*t*) defines the length of follow-up in the *j*-th interval:

$$ \delta_{j}(t)=\left\{\begin{array}{ll} 0 & \text{if~}\, t\leq a_{j-1},\\ t-a_{j-1} & \text{if~}\, a_{j-1}<t\leq a_{j}, \\ a_{j}-a_{j-1} & \text{if~}\, t>a_{j}. \\ \end{array} \right. $$

The so-called population attributable fraction (PAF) estimator [8] is then defined using the following parametric estimators:

$$\begin{aligned} \hat{S}_{0}(t)&=\frac{1}{n}\sum\limits_{i=1}^{n}\hat{S}_{PCH}(t|Z_{i}=0) \text{~and~}\\ \hat{S}(t)&=\frac{1}{n}\sum\limits_{i=1}^{n}\hat{S}_{PCH}(t|Z_{i}= z_{i}). \end{aligned} $$

The model parameter estimates $\hat {\alpha }=(\hat {\alpha }_{1}, \ldots,\hat {\alpha }_{J})$ and $\hat {\beta }$ are obtained by maximum likelihood estimation. The variance of $\hat {A}(t)$ is estimated using the delta method [8].

#### Global approaches over the whole follow-up period

Alternatively to the definition of the AR as a function of time, Spiegelman et al. [10] proposed to estimate a single value in cohort studies:

$$ AR = \frac{\sum\limits_{k=0}^{K} q_{k} (RR_{k} - 1)}{1 + \sum\limits_{k=0}^{K} q_{k} (RR_{k} - 1)} $$

where *RR*_*k*_ and *q*_*k*_, *k*=0,…,*K*, are the relative risk and prevalence in the target population for the *k*th combination of risk factors.

Upon using Cox’s proportional hazards model, the overall AR can be estimated using estimated hazard ratio (HR) for relative risk and person-years for exposure prevalence in the cohort. The asymptotic variance is estimated using the multivariate delta method [10].

In the case of an unadjusted, binary exposure variable, the formula by Spiegelman et al. [10] simplifies into

3 $$ AR = \frac{q (RR - 1)}{1 + q (RR - 1)}  $$

where *q* denotes the exposure prevalence and *RR* the relative risk of exposed relative to nonexposed subjects. This formula resembles the well-known formula used by epidemiologists [1, 2] where *q* is estimated by the proportion of exposed subjects at baseline (instead of exposed person-years over the whole follow-up).

### Simulations

In this work, we considered a single, binary covariate *Z* representing exposure with *Z*=0 and 1 for unexposed and exposed subjects respectively, simulated as a Bernoulli random variable with probability of exposure (*q*) set to 0.25, 0.50 and 0.75. To compare the different approaches for estimating AR, we considered either proportional or nonproportional hazards between the exposed and the unexposed.

For proportional hazards, we used instantaneous hazard functions of the form *λ*(*t*|*Z*)=*λ*_0_(*t*) exp(*β* *Z*) where *β* denotes the regression parameter set to ln(2) or 0, and *λ*_0_(*t*) the baseline hazard function taken from a Weibull distribution with shape parameter *γ* and scale parameter *θ*: *λ*_0_(*t*)=*γ* *θ* ^−*γ*^ *t* ^*γ*−1^, and generated event times from (1/*θ*)[− ln(*U*)/ exp(*β* *Z*)]^1/*γ*^ with *U* uniform on (0,1). We explored situations where the baseline hazard was constant (*γ*=1) or dependent on time, either increasing (*γ*=4/3) or decreasing (*γ*=3/4) with time. The scale parameter *θ* was chosen as a function of the shape parameter *γ* so as to obtain median survival time equal to 15 years for unexposed subjects in all scenarios. We calculated survival functions *S*_0_(.) and *S*(.) as exp{−(*t*/*θ*)^*γ*^} and (1−*q*) exp[−(*t*/*θ*)^*γ*^]+*q* exp[−(*t*/*θ*)^*γ*^ exp(*β*)] respectively and derived theoretical values of AR as a function of time from Eq. (2). For the global AR derived from the simpler approach, theoretical values were obtained as *q*[ exp(*β*)−1]/{1+*q*[ exp(*β*)−1]}.

For nonproportional hazards, we generated event times from *G* ^−1^[− ln(*U*)]/[*λ*_0_ exp(*β* *Z*)] assuming a cumulative hazard function of the form *Λ*(*t*|*Z*)=*G*[*λ*_0_ *t* exp(*β* *Z*)] where *G* is the logarithmic transformation *G*(*t*)= ln(1+2*t*)/2 [7]. Setting *λ*_0_=0.1 yielded a median survival time for unexposed subjects equal to 15 years as in the proportional hazards case. Setting the regression coefficient *β* to ln(2), the HR between the exposed and the unexposed decreased from 2 toward 1 over time. We calculated survival functions *S*_0_(.) and *S*(.) as exp{− ln(1+2*λ*_0_ *t*)/2} and (1−*q*) exp{− ln(1+2*λ*_0_ *t*)/2}+*q* exp{− ln(1+2*λ*_0_ *t* exp(*β*))/2} respectively and derived theoretical values of AR as a function of time from Eq. (2).

We generated censoring times independent of the covariate *Z* and event times *T* from a uniform distribution on [0,*τ*], with *τ* the maximal follow-up time of the study set at 20 years. Depending on scenarios, we obtained censoring percentages around 47-68% (ranges across simulations from 42% to 73%).

We generated 1, 000 data sets of *n*=1, 000 or 10, 000 independent observations and calculated estimators $\hat {A}(.)$ of the AR as a function of time and their associated variances using the four approaches: two non-parametric approaches corresponding to the case where *S*_0_(.) and *S*(.) are both estimated by the Kaplan-Meier method (KM) and to the case where *S*_0_(.) and *S*(.) are estimated by the Kaplan-Meier and the weighted Kaplan-Meier methods, respectively (WKM) [7], one semiparametric approach using Cox’s proportional hazards model (COX) [7], and one parametric approach corresponding to the case where survival functions are estimated assuming piecewise constant hazards (PCH) [8] considering four intervals of 5-year width. In the case where no event was generated in any five-year interval, the simulated dataset was discarded and replaced by a new one. We also considered the simpler approach based on Eq. (3) to estimate a global AR.

Results of the time-dependent approaches are presented at times *t*=*τ*/4, *τ*/2, 3*τ*/4 and *τ* (respectively, 5, 10, 15 and 20 years). For the nonparametric and semiparametric approaches, estimates were obtained at times actually observed in the dataset so we considered values taken at the closest preceding time point. While nonparametric estimations are based on data available until the time of interest, semiparametric and parametric methods use data available over the whole follow-up period. To allow for a fairer comparison under the proportional hazards assumption, we also computed semiparametric and parametric estimators after censoring observation times at either *τ*/4 or *τ*/2. The parametric approach was then based on one or two interval(s) of 5-year width respectively.

For all five approaches, results displayed are the average absolute bias relative to the theoretical value *A*(.), the Sampling Standard Deviation of $\hat {A}(.)$ (SSD), the average Standard Error Estimator of *A*(.) (SEE) and the coverage probability (CP) of the 95% confidence interval (CI) of *A*(.). Although authors [7, 8] have suggested to use the complementary logarithmic transformation ln {1−*A*(.)*black*} to improve coverage probabilities in case of small sample size, this did not notably improve coverage probabilities in our results (data not shown) so results presented are for the untransformed *A*(.).

Simulations were performed using R release 3.0.1. We coded the nonparametric methods using R software and tested the validity of our code by comparing our simulation results with those of the authors using the same parameters [7]. For the semiparametric method [7], we used the R package paf developed by Chen [17]. For the parametric method [8], we used SAS release 9.3 and a set of macros developed by Laaksonen et al. [18]. For the global approach by Spiegelman et al. [10], we used the %par SAS macro developed by the authors.

## Results

### Simulations

We first considered the case of proportional hazards between the exposed and the unexposed with *β*= ln(2) and probability of exposure equal to 0.50, starting with a constant baseline hazard. With a sample size of 1, 000 observations and for the four time-dependent approaches (Table 1, left-hand side), there was more upward bias at the end of follow-up *τ*, especially with the KM method and the WKM method (to a lesser extent), but AR estimators for all methods and time points were virtually unbiased (relative bias <2.5*%*). Variance estimators accurately reflected the true variation and the 95% CIs had proper coverage probabilities, except in *τ* for the two nonparametric methods, where the variance was somewhat underestimated, yielding lower than nominal coverage. Parametric and semiparametric estimators were more precise than nonparametric estimators, particularly at times *τ*/4 and *τ*. Estimators of parameter *β* were unbiased for the semiparametric and parametric approaches (relative bias <0.7*%*, data not shown).

**Table 1:** Simulation results for the estimation of attributable risk *A*(.) under proportional hazards, constant baseline hazard (*γ*=1) with regression parameter *β*= ln(2) and probability of exposure *q*=0.5

Estimation method			*n*=1,000				*n*=10,000			
	Time	*A*(*t*)	Bias	SEE	SSD	CP	Bias	SEE	SSD	CP
KM	*τ*/4	0.284	0.001584	0.052440	0.052591	0.949	−0.000011	0.016622	0.016349	0.944
	*τ*/2	0.240	0.001496	0.039210	0.039099	0.948	0.000235	0.012434	0.012420	0.944
	3 *τ*/4	0.200	0.001100	0.035666	0.035948	0.946	−0.000333	0.011353	0.011354	0.949
	*τ*	0.166	0.004047	0.043238	0.053015	0.912	0.001025	0.017251	0.019598	0.943
WKM	*τ*/4	0.284	0.001594	0.052516	0.052483	0.949	0.000003	0.016613	0.016357	0.946
	*τ*/2	0.240	0.001541	0.039144	0.038926	0.950	0.000285	0.012401	0.012398	0.946
	3 *τ*/4	0.200	0.001093	0.035402	0.035479	0.953	−0.000286	0.011283	0.011297	0.952
	*τ*	0.166	0.002922	0.040635	0.048602	0.902	0.000497	0.016646	0.018245	0.942
COX	*τ*/4	0.284	0.000977	0.038843	0.038208	0.958	−0.000136	0.012292	0.012206	0.956
	*τ*/2	0.240	0.001108	0.033847	0.033524	0.951	0.000006	0.010700	0.010616	0.958
	3 *τ*/4	0.200	0.001031	0.029264	0.028893	0.958	−0.000081	0.009237	0.009253	0.954
	*τ*	0.166	0.002577	0.027146	0.027753	0.946	0.000148	0.008965	0.009087	0.950
PCH	*τ*/4	0.284	0.001356	0.038338	0.038248	0.952	−0.000086	0.012120	0.012209	0.953
	*τ*/2	0.240	0.001372	0.033380	0.033529	0.948	0.000034	0.010543	0.010608	0.952
	3 *τ*/4	0.200	0.001113	0.028804	0.028870	0.957	−0.000081	0.009088	0.009263	0.952
	*τ*	0.166	0.001564	0.025811	0.025420	0.961	−0.000154	0.008105	0.008153	0.952
Simpler	–	0.333	0.000826	0.043356	0.043147	0.952	−0.000209	0.013715	0.013776	0.955

*KM* nonparametric approach based on Kaplan-Meier estimation for *S*(*t*), *WKM* nonparametric approach based on weighted Kaplan-Meier estimation for *S*(*t*), *COX* semiparametric approach, *PCH* parametric approach using a piecewise constant hazards model, *Simpler* simpler approach based on proportion of exposed subjects, *Bias* sampling mean of the difference between $\hat {A}(t)$ and *A*(*t*), *SEE* sampling mean of standard error estimate of *A*(*t*), *SSD* sampling standard deviation of $\hat {A}(t)$, *CP* coverage probability of the 95% Wald confidence interval

When considering samples of size 10, 000 (Table 1, right-hand side), bias decreased in magnitude compared to a sample size of 1, 000 observations (relative bias < 0.7*%* for AR in all methods and <0.04*%* for *β* in the semiparametric and parametric approaches). As expected, precision increased markedly for all methods, by a factor of about $\sqrt {10}$. Moreover, SEEs and SSDs were in closer agreement even at time *τ* with nonparametric methods and all coverage probabilities fell within the 0.940 to 0.960 range.

Similar observations held when considering a decreasing baseline hazard (Table 2). When *γ*=3/4, biases were close to those observed with *γ* = 1 except for a moderate increase for the parametric approach and both sample sizes (relative bias <2.4*%*). Nevertheless coverage probabilities remained satisfactory for this method and the others, except again at the end of follow-up *τ* for the two nonparametric methods and *n*=1, 000 (0.915 and 0.906 for KM and WKM respectively).

**Table 2:** Simulation results for the estimation of attributable risk *A*(.) under proportional hazards, decreasing baseline hazard (*γ*=3/4) with regression parameter *β*= ln(2) and probability of exposure *q*=0.5

Estimation method			*n*=1, 000				*n*=10, 000			
	Time	*A*(*t*)	Bias	SEE	SSD	CP	Bias	SEE	SSD	CP
KM	*τ*/4	0.269	0.001799	0.044659	0.045486	0.940	0.000129	0.014162	0.014200	0.946
	*τ*/2	0.231	0.001217	0.036054	0.036037	0.943	0.000351	0.011437	0.011547	0.946
	3 *τ*/4	0.200	0.001164	0.034218	0.034637	0.948	−0.000204	0.010895	0.010746	0.956
	*τ*	0.176	0.003532	0.041550	0.047835	0.915	0.000299	0.016351	0.019086	0.948
WKM	*τ*/4	0.269	0.001832	0.044713	0.045359	0.942	0.000131	0.014153	0.014197	0.946
	*τ*/2	0.231	0.001283	0.035999	0.035858	0.947	0.000368	0.011408	0.011509	0.947
	3 *τ*/4	0.200	0.001132	0.034004	0.034272	0.950	−0.000193	0.010838	0.010716	0.956
	*τ*	0.176	0.002628	0.039647	0.045615	0.906	0.000116	0.015851	0.017720	0.947
COX	*τ*/4	0.269	0.000957	0.036029	0.035611	0.955	0.000107	0.011401	0.011229	0.955
	*τ*/2	0.231	0.001067	0.031741	0.031499	0.954	0.000129	0.010031	0.009949	0.953
	3 *τ*/4	0.200	0.000972	0.028300	0.028071	0.962	0.000060	0.008937	0.008899	0.949
	*τ*	0.176	0.002177	0.026818	0.027274	0.955	0.000168	0.008790	0.008771	0.956
PCH	*τ*/4	0.269	0.003717	0.035027	0.035896	0.940	0.002630	0.011076	0.011300	0.939
	*τ*/2	0.231	0.002926	0.030819	0.031734	0.945	0.001853	0.009736	0.009995	0.936
	3 *τ*/4	0.200	0.002124	0.027440	0.028260	0.949	0.001247	0.008666	0.008949	0.940
	*τ*	0.176	0.001883	0.025457	0.025679	0.958	0.000621	0.008014	0.008240	0.946
Simpler	–	0.333	0.000814	0.041900	0.041749	0.952	0.000050	0.013257	0.013257	0.947

*KM* nonparametric approach based on Kaplan-Meier estimation for *S*(*t*), *WKM* nonparametric approach based on weighted Kaplan-Meier estimation for *S*(*t*), *COX* semiparametric approach, *PCH* parametric approach using a piecewise constant hazards model, *Simpler* simpler approach based on proportion of exposed subjects, *Bias* sampling mean of the difference between $\hat {A}(t)$ and *A*(*t*), *SEE* sampling mean of standard error estimate of *A*(*t*), *SSD* sampling standard deviation of $\hat {A}(t)$, *CP* coverage probability of the 95% Wald confidence interval

Under an increasing baseline hazard (Table 3), coverage probabilities at *τ* of the two nonparametric estimators worsened with *n*=1, 000 (0.891 and 0.898 for KM and WKM approaches respectively) as a result of increased biases compared to constant and decreasing baseline hazards. Results were otherwise satisfactory and biases for the parametric method were comparable with those obtained under constant baseline hazard.

**Table 3:** Simulation results for the estimation of attributable risk *A*(.) under proportional hazards, increasing baseline hazard (*γ*=4/3) with regression parameter *β*= ln(2) and probability of exposure *q*=0.5

Estimation method			*n*=1, 000				*n*=10, 000			
	Time	*A*(*t*)	Bias	SEE	SSD	CP	Bias	SEE	SSD	CP
KM	*τ*/4	0.299	0.000814	0.064311	0.064377	0.947	−0.000024	0.020388	0.020204	0.956
	*τ*/2	0.250	0.002020	0.043388	0.043169	0.952	0.000210	0.013761	0.013651	0.944
	3 *τ*/4	0.200	0.001174	0.037152	0.037027	0.955	−0.000469	0.011824	0.011798	0.960
	*τ*	0.153	0.007382	0.043968	0.054032	0.891	0.000554	0.018140	0.021081	0.939
WKM	*τ*/4	0.299	0.000805	0.064427	0.064296	0.950	−0.000010	0.020380	0.020196	0.954
	*τ*/2	0.250	0.002055	0.043322	0.042973	0.949	0.000272	0.013722	0.013643	0.947
	3 *τ*/4	0.200	0.001193	0.036838	0.036463	0.962	−0.000410	0.011739	0.011741	0.958
	*τ*	0.153	0.005596	0.040652	0.048586	0.898	0.000055	0.017280	0.019095	0.935
COX	*τ*/4	0.299	0.001207	0.041863	0.040891	0.960	−0.000209	0.013250	0.013076	0.962
	*τ*/2	0.250	0.001321	0.036377	0.035580	0.954	−0.000062	0.011499	0.011341	0.958
	3 *τ*/4	0.200	0.001300	0.030350	0.029672	0.956	−0.000121	0.009572	0.009502	0.965
	*τ*	0.153	0.002791	0.027165	0.028199	0.945	−0.000309	0.009206	0.010402	0.945
PCH	*τ*/4	0.299	−0.000084	0.041594	0.040674	0.961	−0.001831	0.013151	0.013022	0.957
	*τ*/2	0.250	0.000876	0.036176	0.035464	0.956	−0.000759	0.011424	0.011313	0.958
	3 *τ*/4	0.200	0.001462	0.030163	0.029655	0.959	−0.000051	0.009509	0.009485	0.961
	*τ*	0.153	0.002572	0.025716	0.024704	0.961	0.000622	0.008058	0.007962	0.945
Simpler	–	0.333	0.001129	0.044983	0.044481	0.955	−0.000242	0.014226	0.014195	0.957

*KM* nonparametric approach based on Kaplan-Meier estimation for *S*(*t*), *WKM* nonparametric approach based on weighted Kaplan-Meier estimation for *S*(*t*), *COX* semiparametric approach, *PCH* parametric approach using a piecewise constant hazards model, *Simpler* simpler approach based on proportion of exposed subjects, *Bias* sampling mean of the difference between $\hat {A}(t)$ and *A*(*t*), *SEE* sampling mean of standard error estimate of *A*(*t*), *SSD* sampling standard deviation of $\hat {A}(t)$, *CP* coverage probability of the 95% Wald confidence interval

With a lower or greater prevalence of exposure (25% or 75% exposed), coverage probabilities in *τ* for the nonparametric approaches improved but sometimes remained lower than the nominal value despite a sample size of 10, 000 (Additional file 1: Table S1 and Additional file 2: Table S2 for *γ*=1 and *β*= ln(2)). The same general picture held with other values of *γ* (data not shown), except for the parametric approach which showed slightly insufficient (93%) coverage at times < *τ* for *γ*=3/4 and both exposure probabilities 0.25 and 0.75.

Under the same parameters but *β*=0 (Additional file 3: Table S3 for *γ*=1 and 50% exposed), results were similar to those with *β*= ln(2) except for slightly improved coverage probabilities in *τ* for the nonparametric approaches and a sample size of 1, 000.

Under all scenarios with proportional hazards (Tables 1, 2 and 3, Additional file 1: Table S1, Additional file 2: Table S2 and Additional file 3: Table S3), estimators of global AR from the simpler approach were virtually unbiased with satisfactory coverage probabilities. The estimated single values were generally greater than those of time-dependent approaches at any point in time.

When follow-up was stopped at *τ*/4 or *τ*/2, under proportional hazards (data not shown), estimates for the two nonparametric methods were of course identical to those obtained at the same time points with a complete follow-up. SEEs for the semiparametric and parametric methods increased, getting closer to those of nonparametric methods with censoring at *τ*/2 and even closer with censoring at *τ*/4. Coverage probabilities remained satisfactory except for the parametric method under decreasing baseline hazard (*γ*=3/4) where they tended to be lower than the nominal value e.g., 0.935 and 0.918 at *τ*/4 for censoring at *τ*/4, *β*= ln(2) and 50% exposed, and for *n*=1, 000 and *n*=10, 000 respectively.

Finally, when considering nonproportional hazards between the exposed and the unexposed (Table 4, for *β*= ln(2) and 50% exposed), nonparametric methods yielded similar results to those under proportional hazards. However, the semiparametric and parametric approaches that both relied on the proportional hazards assumption performed poorly. With a sample size of 1, 000 observations (Table 4, left-hand side), estimates using the semiparametric approach were biased (relative bias between 7.9 and 32.6*%*) with poor coverage probabilities except at *τ*/2. The parametric approach resulted in even more severe biases (relative bias between 14.6 and 81.6*%*) and poorer coverage probabilities. With *n*=10, 000, bias remained high and became similar with the semiparametric and parametric approaches (between 7.1 and 31.2*%* and between 8.3 and 32% respectively), and coverage deteriorated further as a result of tighter 95% CIs (Table 4, right-hand side). With a lower or greater prevalence of exposure, coverage probabilities with the parametric approach improved at all times but generally remained less than 93% (data not shown).

**Table 4:** Simulation results for the estimation of attributable risk *A*(.) under nonproportional hazards with regression parameter *β*= ln(2) and probability of exposure *q*=0.5

Estimation method			*n*=1, 000				*n*=10, 000			
	Time	*A*(*t*)	Bias	SEE	SSD	CP	Bias	SEE	SSD	CP
KM	*τ*/4	0.181	0.001124	0.045053	0.045787	0.954	0.000289	0.014277	0.014126	0.949
	*τ*/2	0.133	0.001330	0.037581	0.037647	0.953	−0.000029	0.011915	0.012154	0.935
	3 *τ*/4	0.109	0.001211	0.036543	0.036593	0.953	−0.000301	0.011618	0.011608	0.952
	*τ*	0.093	0.002743	0.043713	0.051764	0.933	−0.000888	0.016362	0.019957	0.950
WKM	*τ*/4	0.181	0.001138	0.045090	0.045739	0.954	0.000291	0.014274	0.014130	0.949
	*τ*/2	0.133	0.001347	0.037587	0.037593	0.956	−0.000024	0.011911	0.012151	0.938
	3 *τ*/4	0.109	0.001165	0.036511	0.036518	0.952	−0.000293	0.011612	0.011607	0.956
	*τ*	0.093	0.001685	0.042617	0.049261	0.920	−0.000708	0.016157	0.019107	0.946
COX	*τ*/4	0.181	−0.018761	0.037521	0.037543	0.933	−0.019843	0.011869	0.011939	0.621
	*τ*/2	0.133	0.010548	0.033500	0.033580	0.941	0.009504	0.010588	0.010676	0.847
	3 *τ*/4	0.109	0.023376	0.030960	0.031017	0.879	0.022314	0.009775	0.009879	0.368
	*τ*	0.093	0.030360	0.029427	0.029588	0.830	0.029168	0.009323	0.009456	0.127
PCH	*τ*/4	0.181	0.026479	0.048525	0.049191	0.908	−0.017516	0.011688	0.012080	0.672
	*τ*/2	0.133	0.057418	0.044915	0.045594	0.738	0.011082	0.010391	0.010768	0.806
	3 *τ*/4	0.109	0.070045	0.042342	0.043042	0.607	0.023478	0.009571	0.009936	0.313
	*τ*	0.093	0.075924	0.040403	0.041050	0.525	0.029848	0.009011	0.009360	0.098

*KM* nonparametric approach based on Kaplan-Meier estimation for *S*(*t*), *WKM* nonparametric approach based on weighted Kaplan-Meier estimation for *S*(*t*), *COX* semiparametric approach, *PCH* parametric approach using a piecewise constant hazards model, *Bias* sampling mean of the difference between $\hat {A}(t)$ and *A*(*t*), *SEE* sampling mean of standard error estimate of *A*(*t*), *SSD* sampling standard deviation of $\hat {A}(t)$, *CP* coverage probability of the 95% Wald confidence interval

### Data example

As in our simulations, we used time-on-study rather than attained age as the time-scale after checking that both yielded similar results. Fitting a Weibull distribution to the observed survival data and considering incident invasive breast cancer as the event of interest (i.e., considering time to breast cancer occurrence), the shape (*γ*) and scale (*θ*) parameters were estimated as 1.2 and 178.2 respectively and the corresponding estimated Weibull survival function almost coincided with nonparametric Kaplan-Meier estimate (data not shown). The assumption of proportional hazards between women ever-exposed and those never-exposed to any MHT at baseline seemed appropriate (Schoenfeld residual test, *p*=0.7), with an estimated HR at 1.22 (95% CI, 1.13 to 1.33) for MHT exposure from the Cox model.

**Fig. 1:**
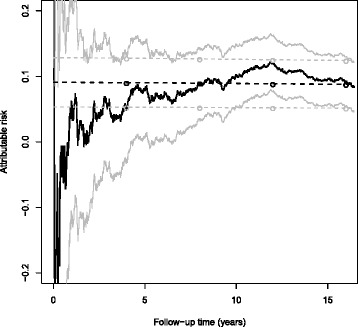
Estimation of the risk of invasive breast cancer attributable to ever use of menopausal hormone therapy at baseline as a time function, E3N cohort, 1992-2008. The *dark solid* and *dark dashed* curves pertain to the point estimates by KM and COX, respectively, the *dark circles* to the point estimates by the 4-year interval PCH; the *light solid* and *light dashed* curves, as well as the *light circles*, show the corresponding 95% confidence intervals. The WKM curves are not displayed because they almost coincided with the KM curves at the chosen scale

The AR estimates from nonparametric approaches KM and WKM were almost identical (Fig. 1). They tended to increase until 12 years of follow-up (e.g., for the KM approach, from 5.5*%* (95% CI, −2.7 to 13.6%) after four years to 12.0*%* (95% CI, 7.8 to 16.2%) after 12 years of follow-up), then to decrease and converge to semiparametric and parametric estimates at the end of follow-up with an estimated 9.2*%* AR (95% CI, 5.4 to 13.0%) after 16 years. In comparison, estimates using the semiparametric and parametric approaches slightly decreased monotonically over time from 9.0*%* (95% CI, 5.3 to 12.8%) to 8.8*%* (95% CI, 5.1 to 12.4%) and from 8.9*%* (95% CI, 5.2 to 12.6%) to 8.7*%* (95% CI, 5.0 to 12.3%) respectively. Thus, after 16 years of follow-up, the proportion of invasive breast cancer cases attributable to MHT exposure was close to 9% whatever the method used. Estimates using nonparametric approaches were far less precise at earlier times and displayed wider 95% CIs (even including 0 at time 4 years) than semiparametric and parametric approaches in the first half of the follow-up: e.g., at time 8 years, AR was estimated as 8.9*%* (95% CI, 3.5 to 14.4*%*) and 9.0*%* (95% CI, 5.2 to 12.7*%*) from the KM and Cox approaches, respectively. Adjusting for age at baseline, either as a continuous covariate in the semiparametric approach or as a dichotomous covariate in all approaches, hardly modified these estimates (data not shown).

Finally, using the method proposed by Spiegelman et al. [10], we found that 9.2% (95% CI, 5.4 to 13.0%) of cases who developed invasive breast cancer at various times in the cohort follow-up were attributable to MHT exposure. Using the simpler approach with the proportion of exposed subjects at inclusion, we obtained a close, slightly smaller estimate at 9.1% (95% CI, 5.3 to 12.8%).

## Discussion

Comparing different methods of AR estimation when disease incidence is interpreted as a CDF [7, 8], we observed that AR estimators were essentially unbiased for all approaches when we generated event times from a proportional hazards model. Empirical and estimated variances were close, with proper coverage probabilities except at the end of follow-up for the nonparametric methods and a smaller sample size. When considering a non-constant baseline hazard, estimates using the parametric approach were robust despite misspecification of the baseline hazard. For nonparametric approaches, biases tended to increase at the end of follow-up (time *τ*) when the baseline hazard increased with time. With the simpler approach, results were satisfactory. However, under nonproportional hazards, estimates using the semiparametric and parametric approaches were biased with poor coverage probabilities.

To our knowledge, this is the first simulation study comparing nonparametric, semiparametric and parametric methods of AR estimation as a function of time as well as a simpler, global approach for a diversity of scenarios (proportional or nonproportional hazards, constant or nonconstant baseline hazard, varying exposure probabilities, strengths of association and sample sizes) in the survival analysis context. Chen et al. [7] reported simulations for the Kaplan-Meier, weighted Kaplan-Meier and transformation models when event times were generated from proportional or nonproportional hazards models with regression parameter *β*=1, 40% probability of exposure and a sample size of 1, 000 observations. Like them, we found that, under the assumption of independent censoring, results with KM and WKM approaches were very close. Differences between the two nonparametric approaches were apparent when censoring was dependent on covariates [7], which we did not evaluate in this study.

Also in line with Chen et al. [7], when we generated event times from a proportional hazards model, we found that nonparametric and semiparametric estimates were all unbiased, nonparametric estimates had larger variances than semiparametric estimates and estimated variances accurately reflected the true variance except in *τ* for the nonparametric approaches and a sample size of 1, 000 observations. Nonparametric approaches tended to perform better (respectively worse) when exposure prevalence was lower (respectively higher) which could be expected from the possibly unstable and inefficient Kaplan-Meier estimator of survival among the unexposed when the proportion of those is small [7]. This general picture held in our simulations whether event times were generated with constant, decreasing or increasing baseline hazard. We note, however, that, when we considered a larger sample size, the discrepancies between estimated and empirical variances tended to diminish, with most often satisfactory coverage probabilities in *τ*.

For nonproportional hazards, we generated event times using a transformation model considered by Chen et al. [7] and found consistent results for the nonparametric approaches, similar to those in the case of proportional hazards. However, while Chen et al. [7] applied the same nonproportional hazards model for both data generation and analysis (AR estimation), we generated data under nonproportional hazards and estimated AR from (misspecified) Cox’s proportional hazards model. This explains the impaired performance we observed when the proportional hazards assumption was violated in contrast with the satisfactory results obtained by Chen et al. [7]. Sjölander and Vansteelandt [9] recently proposed an alternative semiparametric estimator of AR also based on Cox’s proportional hazards model that proved robust to various model misspecifications. However these authors did not evaluate deviations from the proportional hazards assumption.

Like Chen et al. [7] in their simulation and example analysis, we observed greater imprecision of the nonparametric estimators at the start of follow-up, which could explain possible early negative AR values. This imprecision could be expected because the estimation of the survival function relies upon the information available until the time of interest and not many events have yet occurred by then. This differs from the semiparametric and parametric methods which take advantage of the estimation of parameter *β* being performed over the entire follow-up. Consistently, we found larger variances for the semiparametric and parametric approaches with shorter lengths of follow-up.

Another novelty of this work was the evaluation of the parametric approach to AR estimation proposed by Laaksonen et al. [8] using simulations and its comparison with nonparametric and semiparametric approaches. Generally under proportional hazards, we found close agreement between the semiparametric and parametric approaches, in our simulations as well as in the example. Of note, the parametric approach seemed robust despite misspecification of baseline hazard, i.e., when we considered decreasing or increasing (instead of constant) baseline hazard and proportional hazards. However, like the semiparametric approach based on Cox’s model, the parametric approach was sensitive to the proportional hazards assumption and performed poorly in our simulations when this assumption was violated. We also evaluated the simpler, global approach and our results were satisfactory under proportional hazards.

As noted by several authors [4, 7], simpler approaches based on Eq. (1) or equivalent formulas [1, 2] are generally defined for binary outcomes with time-independent risk factors. Consequently, they prove to be inadequate for cohort studies with censored time-to-event outcomes and possibly time-dependent covariates. In contrast, the nonparametric, semiparametric and parametric approaches we considered here have been specifically developped for censored time-to-event outcomes and produce AR estimate as a function of time, thus allowing the AR to be time-varying. A major limitation of the simpler approach in the context of cohort studies is that it only takes account of the proportion of exposed subjects at the beginning of follow-up. The proportion of exposed subjects indeed decreases as follow-up time increases (because exposed subjects fail earlier than nonexposed subjects) [6]. This explains why our AR estimates from the simpler approach were generally greater than those from time-dependent approaches and further underlines why approaches estimating AR as a function of time are an improvement on the simpler approach in the context of survival analysis.

In our study, we used the definition of AR based on CDFs because it is a natural extension of the standard AR definition (Eq. (1)) for time-to-event outcomes [6–9] and it is equivalent to the standard definition when time *t* is the end of follow-up in cohort studies [4]. In addition several estimation methods have been proposed for the CDF-based AR definition in cohort studies and the survival analysis context in contrast to the alternative definition based on instantaneous hazard functions [4, 5] for which only one method of estimation based on Cox’s proportional hazards model has been published [4].

In cohort studies where exposed individuals are over-sampled relative to the exposure prevalence in the population, AR will correctly reflect the impact of exposure in the cohort, but the impact at the population level will be overestimated. The marginal survival function *S*(*t*) should be corrected in order to alleviate this upward bias on AR estimates. The AR (and its estimates) being a function of time, various representations of AR estimates can be used. We used a graphical representation of the whole time function in our example and produced estimates at four equally spaced times in our simulations. Alternatively, a single overall estimate could be obtained by averaging out the time function of AR estimates or by using the alternative approach by Spiegelman et al. [10] as in our example.

We chose our simulation parameters to resemble real epidemiologic cohorts. These often include a few thousands participants followed for several years. For a smaller sample size (*n*=500), whether we used the logarithmic transformation or not, we observed findings generally similar to those presented with a sample size of 1,000 observations. This was true with the notable exception of the less than nominal coverage probability for the semiparametric approach at time *τ* for constant (*γ*=1) and decreasing (*γ*=3/4) baseline hazards, and at times *τ*/4 and *τ* for increasing (*γ*=4/3) baseline hazard (data not shown).

In our application, the proportional hazards assumption seemed appropriate, as well as a Weibull distribution for event times with an increasing baseline hazard and shape parameter halfway between the values *γ*=1 and 4/3 considered in our simulation study. Exposure frequency was also close to our simulated 0.5 probability of exposure. However, as in many epidemiologic cohorts, the censoring rate was much greater in our example (94.2*%*) than in our simulations. The resulting imprecision may explain the nonparametric AR estimates apparently increasing until three quarters of total follow-up but compatible with the more expected decreasing trend. Chen et al. [7] observed the same finding in their application on a shorter length of follow-up.

Using the approach described by Spiegelman et al. [10], the overall AR estimate for ever use of MHT at baseline was 9.2% in the E3N cohort. This estimate was close to those obtained at the end of follow-up with the nonparametric methods and at the start of follow-up with the parametric and semiparametric approaches. In a recent publication, Dartois et al. [12] reported a higher AR estimate of 14.5% (95% CI, 9.2 to 19.6%) for recent MHT use and postmenopausal invasive breast cancer from the E3N cohort data, using the approach proposed by Spiegelman et al. [10] and a more refined, adjusted analysis with MHT exposure as a time-dependent covariate.

This study has some limitations. First, we did not evaluate AR estimates adjusted for covariates. Adjustment for multiple variables is common practice in epidemiology, especially age which can also be used as the underlying time-variable [19]. In our example, using analyses unadjusted or parametrically adjusted for age, there was a statistically significant association between baseline MHT ever use and breast cancer risk, in line with findings from more complex models with age as the timescale and adjustment for other covariates in the original study [11]. Although adjustment for covariates is available in packages for semiparametric and parametric approaches [7, 8], there are constraints in nonparametric approaches as the number of covariates must be limited and adjustment for continuous variables is not possible. Moreover, available packages for estimating the AR would need to be adapted to allow left truncation resulting from using age as the timescale. Second, in our example, we only considered women who had ever received MHT at baseline as exposed whereas exposure can vary during follow-up. Other methodological studies are needed to take into account the exposure time dependency for estimating AR as a function of time [9]. Finally, we have ignored the competing risk of death and cancers of other sites (11.2% of our 94.2% censored observations) which might also bias our estimate of breast cancer risk attributable to MHT [20].

## Conclusions

The AR estimators from the four time-dependent methods had satisfactory performance under the proportional hazards assumption. Estimators using semiparametric and parametric approaches were not robust in case of nonproportional hazards. Lack of precision could be an issue for nonparametric methods at the beginning of the follow-up time in cohorts of relatively low sample size. In practice, if the proportional hazards assumption seems appropriate, the semiparametric or parametric approaches should be used.

## Additional files

Additional file 1 Simulation results for the estimation of attributable risk *A*(.) under proportional hazards, constant baseline hazard (*γ*=1) with regression parameter *β*= ln(2) and probability of exposure *q*=0.25. (PDF 20.3 kb)

Additional file 2 Simulation results for the estimation of attributable risk *A*(.) under proportional hazards, constant baseline hazard (*γ*=1) with regression parameter *β*= ln (2) and probability of exposure *q*=0.75. (PDF 20.5 kb)

Additional file 3 Simulation results for the estimation of attributable risk *A*(.) under proportional hazards, constant baseline hazard (*γ*=1) with regression parameter *β*=0 and probability of exposure *q*=0.5. (PDF 20.1 kb)

## Abbreviations

AR: Attributable risk
CDF: Cumulative Distribution Function
CI: Confidence Interval
COX: Cox’s proportional hazards model
CP: Coverage Probability
E3N: *Étude Épidémiologique auprès de Femmes de la Mutuelle Générale de l’Éducation Nationale*
HR: Hazard Ratio
KM: Kaplan-Meier
MHT: Menopausal Hormone Therapy
PCH: Piecewise Constant Hazards
RR: Relative Risk
SEE: Standard Error Estimator
SSD: Sampling Standard Deviation
WKM: Weighted Kaplan-Meier

## References

[1] Cole P, MacMahon B (1971). Attributable risk percent in case-control studies. Br J Prev Soc Med.

[2] Levin ML (1953). The occurrence of lung cancer in man. Acta Unio Int Contra Cancrum.

[3] Bénichou J (2001). A review of adjusted estimators of attributable risk. Stat Methods Med Res.

[4] Chen YQ, Hu C, Wang Y (2006). Attributable risk function in the proportional hazards model for censored time-to-event. Biostatistics.

[5] Samuelsen SO, Eide GE (2008). Attributable fractions with survival data. Stat Med.

[6] Cox C, Chu H, Muñoz A (2009). Survival attributable to an exposure. Stat Med.

[7] Chen L, Lin DY, Zeng D (2010). Attributable fraction functions for censored event times. Biometrika.

[8] Laaksonen MA, Knekt P, Härkänen T, Virtala E, Oja H (2010). Estimation of the population attributable fraction for mortality in a cohort study using a piecewise constant hazards model. Am J Epidemiol.

[9] Sjölander A, Vansteelandt S. Doubly robust estimation of attributable fractions in survival analysis. Stat Methods Med Res. 2014. (in press). doi:10.1177/0962280214564003. 10.1177/0962280214564003 25519888

[10] Spiegelman D, Hertzmark E, Wand HC (2008). Point and interval estimates of partial population attributable risks in cohort studies: examples and software. Cancer Causes Control.

[11] Fournier A, Mesrine S, Dossus L, Boutron-Ruault MC, Clavel-Chapelon F, Chabbert-Buffet N (2014). Risk of breast cancer after stopping menopausal hormone therapy in the E3N cohort. Breast Cancer Res Treat.

[12] Dartois L, Fagherazzi G, Boutron-Ruault MC, Delaloge S, Mesrine S, Clavel-Chapelon F, Baglietto L (2016). Proportion of premenopausal and postmenopausal breast cancers attributable to known risk factors: Estimates from the E3N-EPIC cohort. Int J Cancer.

[13] Kaplan EL, Meier P (1958). Non parametric estimation from incomplete observations. J Am Stat Assoc.

[14] Murray S, Tsiatis AA (1996). Nonparametric survival estimation using prognostic longitudinal covariates. Biometrics.

[15] Cox DR (1972). Regression models and life tables (with discussion). J R Stat Soc Series B.

[16] Breslow NE (1972). Discussion of the paper by D. R. Cox. J R Stat Soc Series B.

[17] Chen L, ‘paf’. Attributable fraction function for censored survival data, R package version 1.0. 2014. http://cran.r-project.org/web/packages/paf/index.html. Accessed 2 June 2014.

[18] Laaksonen MA, Virtala E, Knekt P, Oja H, Härkänen T (2011). SAS macros for calculation of population attributable fraction in a cohort study design. J Stat Softw.

[19] Thiébaut ACM, Bénichou J (2004). Choice of time-scale in Cox’s model analysis of epidemiologic cohort data: a simulation study. Stat Med.

[20] Laaksonen MA, Härkänen T, Knekt P, Virtala E, Oja H (2010). Estimation of population attributable fraction (PAF) for disease occurrence in a cohort study design. Stat Med.

