# Supplementary material for: Comparison of methods for estimating the attributable risk in the context of survival analysis
Source: BMC Med Res Methodol. 2017 Jan 23;17:10. doi: 10.1186/s12874-016-0285-1 (PMC5259851; doi:10.1186/s12874-016-0285-1)
Supplement: Additional file 1 — Simulation results for the estimation of attributable risk A(.) under proportional hazards, constant baseline hazard (γ=1) with regression parameter β= ln(2) and probability of exposure q=0.25. (PDF 20.3 kb) [file 12874_2016_285_MOESM1_ESM.pdf]

Table S1 Simulation results for the estimation of attributable risk $A(.)$ under proportional hazards, constant baseline hazard ($\gamma = 1$) with regression parameter $\beta = \ln(2)$ and probability of exposure $q = 0.25$

Estimation method	Time	$A(t)$	$n = 1{,}000$ Bias	SEE	SSD	CP	$n = 10{,}000$ Bias	SEE	SSD	CP
KM	$\tau/4$	0.166	0.000767	0.037566	0.037107	0.957	−0.000344	0.011889	0.011883	0.954
	$\tau/2$	0.136	0.000318	0.026562	0.026090	0.954	−0.000062	0.008412	0.008505	0.948
	$3\tau/4$	0.111	0.000345	0.022928	0.022308	0.958	−0.000162	0.007283	0.007317	0.951
	τ	0.090	0.000608	0.027222	0.033360	0.921	0.000311	0.010786	0.012074	0.951
WKM	$\tau/4$	0.166	0.000772	0.037668	0.036910	0.959	−0.000311	0.011871	0.011871	0.954
	$\tau/2$	0.136	0.000310	0.026405	0.025910	0.961	−0.000018	0.008350	0.008425	0.947
	$3\tau/4$	0.111	0.000490	0.022436	0.022317	0.954	−0.000149	0.007161	0.007210	0.955
	τ	0.090	0.000011	0.023394	0.027320	0.908	0.000035	0.009801	0.010984	0.926
COX	$\tau/4$	0.166	0.000446	0.028454	0.027789	0.962	−0.000238	0.009000	0.009034	0.950
	$\tau/2$	0.136	0.000327	0.022835	0.022332	0.957	−0.000132	0.007217	0.007226	0.952
	$3\tau/4$	0.111	0.000112	0.018279	0.017625	0.962	−0.000202	0.005767	0.005767	0.953
	τ	0.090	0.000947	0.015938	0.016030	0.947	−0.000031	0.005241	0.005310	0.950
PCH	$\tau/4$	0.166	0.000672	0.027455	0.027806	0.956	−0.000210	0.008677	0.009038	0.938
	$\tau/2$	0.136	0.000474	0.021919	0.022349	0.950	−0.000116	0.006924	0.007227	0.940
	$3\tau/4$	0.111	0.000145	0.017434	0.017630	0.953	−0.000208	0.005502	0.005761	0.944
	τ	0.090	0.000344	0.014600	0.014513	0.952	−0.000189	0.004577	0.004667	0.949
Simpler	–	0.200	0.000623	0.033982	0.034253	0.955	−0.000295	0.010748	0.011128	0.939

KM nonparametric approach based on Kaplan-Meier estimation for $S(t)$,
WKM nonparametric approach based on weighted Kaplan-Meier estimation for $S(t)$,
COX semiparametric approach, *PCH* parametric approach using a piecewise constant hazards model,
Simpler simpler approach based on proportion of exposed subjects,
Bias sampling mean of the difference between $\hat{A}(t)$ and $A(t)$,
SEE sampling mean of standard error estimate of $A(t)$,
SSD sampling standard deviation of $\hat{A}(t)$,
CP coverage probability of the 95% Wald confidence interval
