# Supplementary material for: Comparison of methods for estimating the attributable risk in the context of survival analysis
Source: BMC Med Res Methodol. 2017 Jan 23;17:10. doi: 10.1186/s12874-016-0285-1 (PMC5259851; doi:10.1186/s12874-016-0285-1)
Supplement: Additional file 2 — Simulation results for the estimation of attributable risk A(.) under proportional hazards, constant baseline hazard (γ=1) with regression parameter β= ln (2) and probability of exposure q=0.75. (PDF 20.5 kb) [file 12874_2016_285_MOESM2_ESM.pdf]

Table S2 Simulation results for the estimation of attributable risk $A(.)$ under proportional hazards, constant baseline hazard ($\gamma = 1$) with regression parameter $\beta = \ln(2)$ and probability of exposure $q = 0.75$

Estimation			$n = 1{,}000$				$n = 10{,}000$			
method	Time	$A(t)$	Bias	SEE	SSD	CP	Bias	SEE	SSD	CP
KM	$\tau/4$	0.373	−0.002134	0.075473	0.075167	0.956	0.000578	0.023935	0.024026	0.953
	$\tau/2$	0.321	−0.001817	0.058878	0.058752	0.944	0.000143	0.018721	0.018706	0.960
	$3\tau/4$	0.273	−0.001380	0.055677	0.058463	0.933	0.000299	0.017818	0.017566	0.950
	τ	0.229	0.002988	0.064332	0.079263	0.880	−0.000864	0.026996	0.033048	0.923
WKM	$\tau/4$	0.373	−0.002145	0.075537	0.075176	0.956	0.000561	0.023933	0.024012	0.953
	$\tau/2$	0.321	−0.001797	0.058865	0.058784	0.943	0.000131	0.018709	0.018715	0.959
	$3\tau/4$	0.273	−0.001410	0.055508	0.058293	0.933	0.000328	0.017795	0.017551	0.948
	τ	0.229	0.002194	0.063226	0.077751	0.879	−0.000705	0.026799	0.032880	0.919
COX	$\tau/4$	0.373	−0.002641	0.054982	0.055028	0.948	0.000035	0.017371	0.017306	0.955
	$\tau/2$	0.321	−0.001861	0.050909	0.050975	0.947	0.000099	0.016102	0.016095	0.952
	$3\tau/4$	0.273	−0.001044	0.046584	0.046373	0.952	0.000180	0.014736	0.014743	0.949
	τ	0.229	0.000817	0.043942	0.045675	0.944	−0.000077	0.014623	0.015048	0.951
PCH	$\tau/4$	0.373	−0.002187	0.054875	0.055066	0.949	0.000104	0.017318	0.017314	0.952
	$\tau/2$	0.321	−0.001530	0.050815	0.050969	0.946	0.000130	0.016052	0.016088	0.951
	$3\tau/4$	0.273	−0.000930	0.046465	0.046375	0.951	0.000192	0.014684	0.014709	0.948
	τ	0.229	−0.000619	0.042921	0.043412	0.944	0.000102	0.013576	0.013704	0.948
Simpler	–	0.429	−0.003536	0.058323	0.058297	0.951	−0.000106	0.018358	0.018324	0.952

KM nonparametric approach based on Kaplan-Meier estimation for $S(t)$,
WKM nonparametric approach based on weighted Kaplan-Meier estimation for $S(t)$,
COX semiparametric approach, *PCH* parametric approach using a piecewise constant hazards model,
Simpler simpler approach based on proportion of exposed subjects,
Bias sampling mean of the difference between $\hat{A}(t)$ and $A(t)$,
SEE sampling mean of standard error estimate of $A(t)$,
SSD sampling standard deviation of $\hat{A}(t)$,
CP coverage probability of the 95% Wald confidence interval
