# Supplementary material for: Comparison of methods for estimating the attributable risk in the context of survival analysis
Source: BMC Med Res Methodol. 2017 Jan 23;17:10. doi: 10.1186/s12874-016-0285-1 (PMC5259851; doi:10.1186/s12874-016-0285-1)
Supplement: Additional file 3 — Simulation results for the estimation of attributable risk A(.) under proportional hazards, constant baseline hazard (γ=1) with regression parameter β=0 and probability of exposure q=0.5. (PDF 20.1 kb) [file 12874_2016_285_MOESM3_ESM.pdf]

Table S3 Simulation results for the estimation of attributable risk $A(.)$ under proportional hazards, constant baseline hazard ($\gamma = 1$) with regression parameter $\beta = 0$ and probability of exposure $q = 0.5$

Estimation method	Time	$A(t)$	$n = 1{,}000$ Bias	SEE	SSD	CP	$n = 10{,}000$ Bias	SEE	SSD	CP
KM	$\tau/4$	0	0.001588	0.066720	0.066661	0.951	0.000298	0.021095	0.020829	0.959
	$\tau/2$	0	0.001831	0.049184	0.049848	0.945	0.000730	0.015565	0.015568	0.940
	$3\tau/4$	0	0.000775	0.044459	0.044906	0.948	−0.000044	0.014117	0.014006	0.950
	τ	0	0.003632	0.054707	0.063368	0.921	0.001753	0.021781	0.024252	0.955
WKM	$\tau/4$	0	0.001584	0.066767	0.066675	0.951	0.000300	0.021097	0.020840	0.959
	$\tau/2$	0	0.001849	0.049203	0.049846	0.946	0.000731	0.015568	0.015582	0.940
	$3\tau/4$	0	0.000670	0.044428	0.044884	0.948	−0.000039	0.014115	0.014006	0.951
	τ	0	0.002958	0.052653	0.060895	0.905	0.001605	0.021354	0.024770	0.948
COX	$\tau/4$	0	0.001693	0.047709	0.047293	0.961	0.000334	0.015079	0.014993	0.958
	$\tau/2$	0	0.001521	0.042265	0.041903	0.963	0.000294	0.013349	0.013276	0.958
	$3\tau/4$	0	0.001314	0.037280	0.036941	0.963	0.000261	0.011758	0.011698	0.958
	τ	0	0.001276	0.032920	0.032690	0.962	0.000222	0.010319	0.010275	0.958
PCH	$\tau/4$	0	0.001755	0.047745	0.047311	0.961	0.000335	0.015080	0.014992	0.959
	$\tau/2$	0	0.001581	0.042294	0.041918	0.962	0.000295	0.013349	0.013274	0.959
	$3\tau/4$	0	0.001378	0.037290	0.036950	0.962	0.000262	0.011757	0.011694	0.959
	τ	0	0.001248	0.032792	0.032573	0.963	0.000221	0.010312	0.010254	0.959
Simpler	–	0	0.043998	0.053626	0.033774	0.961	0.013205	0.016996	0.009928	0.957

KM nonparametric approach based on Kaplan-Meier estimation for $S(t)$,
WKM nonparametric approach based on weighted Kaplan-Meier estimation for $S(t)$,
COX semiparametric approach, *PCH* parametric approach using a piecewise constant hazards model,
Simpler simpler approach based on proportion of exposed subjects,
Bias sampling mean of the difference between $\hat{A}(t)$ and $A(t)$,
SEE sampling mean of standard error estimate of $A(t)$,
SSD sampling standard deviation of $\hat{A}(t)$,
CP coverage probability of the 95% Wald confidence interval
